# Preparation and Characterization of Macroalgae Biochar Nanomaterials with Highly Efficient Adsorption and Photodegradation Ability

**DOI:** 10.3390/ma11091709

**Published:** 2018-09-13

**Authors:** Yarui Zhou, Hailong Zhang, Lu Cai, Jian Guo, Yaning Wang, Lili Ji, Wendong Song

**Affiliations:** 1 School of Naval Architecture and Mechanical-electrical Engineering, Zhejiang Ocean University, Zhoushan 316022, China; zyr0612Z@gmail.com; 2 Institute of Innovation & Application, Zhejiang Ocean University, Zhoushan 316022, China; zhanghailong@zjou.edu.cn (H.Z.); wangyaning@zjou.edu.cn (Y.W.); 3 College of Environmental and Science Technology, Donghua University, Shanghai 201620, China; cailu@zjou.edu.cn; 4 College of Food and Medical, Zhejiang Ocean University, Zhoushan 316022, China; guojian@zjou.edu.cn; 5 College of Petrochemical and Energy Engineering, Zhejiang Ocean University, Zhoushan 316022, China

**Keywords:** nanomaterial, kelp biochar, adsorption capacity, photocatalytic activity, removal efficiency

## Abstract

In this study, carbonized kelp biochar (AKB) modified by KOH impregnation and photocatalytic Bi_2_MoO_6_/AKB composite (BKBC) nanomaterials were the first time successfully synthesized for efficient removal of dyes in aqueous solution. BET, XRD, FT-IR, and SEM were employed to characterize as-prepared samples. UV-vis and other test results indicated that the removal efficiency of methylene blue (MB) was 61.39% and 94.12% for BKBC and AKB, respectively, which was up to 13 times and 20 times higher in comparison with pure Bi_2_MoO_6_ (PBM). In addition, the equilibrium adsorption capacity of MB could reach up to 324.1 mg/g for AKB. This high dyes adsorption performance could be likely attributed to its high specific surface area (507.177 m^2^/g) and its abundant presence of various functional groups such as –OH and =C–H on AKB. Particularly, the existing of amorphous carbon and transition metal oxides, such as Fe_2_O_3_ and Mn_5_O_8_, could be beneficial for the photodegradation of MB for AKB. Meanwhile, experimental data indicated that adsorption kinetics complied with the pseudo-second order model well, and all of the tests had satisfactory results in terms of the highly efficient adsorption and photodegradation activity of AKB nanomaterials, which suggested its great potential in wastewater treatment.

## 1. Introduction

As methylene blue (MB) was widely applied in the fields of dyes, drugs, chemical indicators, and other fields, discharge of coloured waste without proper treatment could become serious water pollution problems in the environment [1,2]. Discharge of dye could cause the transmittance of water to decrease, which would result in numerous problems in the water system, even at low concentrations [3]. Several methods had been applied to degrade dyes including chemical oxidation, microbiological degradation, electrochemical treatment, adsorption, photocatalytic degradation, etc. [4,5,6,7]. Particularly, adsorption technology was one of the most attractive methods among them [8].

Nanoporous activated carbons have attracted considerable attention in many environmental areas because of the hydrophobic nature of their surface, large surface area, large pore volume, as well as mechanical and thermal stability [9,10]. Biomaterials, especially macroalgae, have drawn much research awareness for the production of carbon nanostructure with a good adsorption performance, because of their high carbon content, high reproduction rate, and low price [11,12]. Utilization of algae carbon as an adsorbent, supercapacitor, or biobriquettes has been well addressed. Rathinam coworkers [13] has carried out research on the adsorption of phenol onto activated carbon prepared from seaweed. The kinetics and equilibrium of adsorption of basic blue dye by green macroalgae have been studied and analyzed [14]. Yang et al. explored the adsorption properties of fluoride on modification of carbon derived from *Sargassum* sp. by lanthanum [15]. Seaweeds have been reported to remove elemental mercury (Hg^0^) from flue gas by Liu et al., which were used to prepare bio-chars first via pyrolysis process and then modification by halides reagents while using a simple impregnation method [16]. Nautiyal et al. utilized the residual biomass of *Spirulina platensis* to remove up to 82.6% of Congo red (CR) dye [17]. Zheng et al. suggested that biochar from *Chlorella* sp. Cha-01 had a high potential to remove pnitrophenols in wastewater treatment [18]. Other studies on the preparation of seaweeds carbon using as electrode materials and biobriquettes have been recorded [19,20,21,22,23,24]. However, the utilization of macroalgae for the preparation of photocatalysis and carrier material has not been reported elsewhere.

The aim of this study was to develop novel mesoporous kelp biochar nanomaterials to assess its adsorption and photodegradation ability of as-prepared samples for the removal of MB in aqueous solution. In this paper, we demonstrated that activated kelp biochar (AKB) via abundant and local available kelp was first-time successfully prepared through pyrolysis at 800 °C and then by chemical activation method while using KOH as an activating agent. Also, a novel Bi_2_MoO_6_/AKB nanocomposite (BKBC) was fabricated by a combination of pyrolysis and a solvothermal technique. The adsorption kinetics and photocatalytic activity of as-prepared samples for removal of MB were investigated. In addition, the structure, morphology, and photocatalytic properties of the synthesized samples were characterized by XRD, SEM, UV-Vis DRS, and FT-IR. The unique features of AKB indicated that it may have a great potential and a high expectation in the practical application to remove the organic pollutants in waste water.

## 2. Materials and Methods

### 2.1. Materials

Fresh kelp was obtained from Zhou Shan, Zhe Jiang, China. Kelp was washed with water several times and then dried at 73 °C for 48 h prior to use. Dried kelp were ground and sieved to a particle size of 100 mesh, and this particle size was used in the following pyrolysis experiments. Bi(NO_3_)·5H_2_O, Na_2_MoO_4_·H_2_O, Methylene blue (MB, C_16_H_18_N_3_SCl), ethylene glycol (C_2_H_6_O_2_ ≥ 99%), ethanol (C_2_H_6_O > 99.7%), and hydrochloric acid (HCl, 36~38%) were purchased from Sinopharm Chemical Reagent Co., Ltd., Shanghai, China. Chemicals that were used in this study were analytical grade and were used as received without further purification.

### 2.2. Preparation of Activated Kelp Biochar (AKB)

45 g of the dried and sieved kelp powder was pre-carbonized in a tubular furnace under nitrogen flow (100 mL min^−1^) up to 800 °C with a rate of 10 °C min^−1^, and the final temperature was on hold for 3 h. The pre-carbonized kelp biochar mixed with KOH (KOH weight/carbon weight = 3:1) and then activated in that tubular furnace at 800 °C for 2 h under the same conditions. This cooled mixture was washed with 0.1 mol/L HCl solution to achieve a pH of 7 in a water bath at 80 °C, then filtered, and washed with deionized water three times. The washed product was further dried in an oven at 73 °C for a night and stored in air-tight sample vessel, and the as-prepared samples were denoted as AKB.

### 2.3. Fabrication of Bi_2_MoO_6_–AKB Composite (BKBC)

Bi_2_MoO_6_/AKB composite was synthesized by a solvothermal technique. In a typical case, 0.9144 g AKB was dispersed into 20 mL ethanol under magnetic stirring [25]. Then, 0.363 g Bi(NO_3_)_3_·5H_2_O and 0.0907 g Na_2_MoO_4_·2H_2_O were dissolved in 7 mL of ethylene glycol under magnetic stirring for 30 min, respectively, followed by two solutions slowly added drop-wise into the above solution under magnetic stirring for 12 h [26,27]. The resulting mixtures were heated to 160 °C and maintained for 24 h in a 50 mL Teflon-lined stainless steel autoclave (KH-50 mL, Gongyi Yuhua Instrument Factory, He Nan, China). After the sample cooled down to room temperature, it was then washed with deionized water and ethanol to remove any ionic residual, and then dried in an oven at 73 °C for 12 h. Finally, the as-fabricated sample was denoted as BKBC. For comparison, pure Bi_2_MoO_6_ (PBM) was prepared by adopting the method in the absence of AKB [28,29].

### 2.4. Characterization

Scanning electron microscopy (SEM Hitachi S-4800, Tokyo, Japan) was employed to investigate microstructures and morphology of the samples. An elemental analyzer (G4ICARUS, Bruker, Karlsruhe, Germany) was manipulated to measure the C, H, and N contents in AKB. Meanwhile, inductively coupled plasma-optical emission spectroscopy (ICP-OES, 5300DV, PerkinElmer, Waltham, Massachusetts USA) was also set to evaluate the chemical composition of the samples. X-ray diffraction (XRD) patterns of the samples were recorded on an Ultima IV X-ray Diffractometer (Ultima IV, Rigaku Corporation, Tokyo, Japan) in the range of 2θ from 20 to 80°. The UV–vis diffuse reflectance spectra were obtained by using UV–Vis spectrophotometer (model no: UV 2600, Shimadzu, Shanghai (branch office), China) in the wavelength range of 200–1000 nm at room temperature. Moreover, surface chemical functional groups were measured by Perkin Elmer Fourier transform infrared (FT-IR, Nicoletteis 50, Thermo Fourier, Waltham, Massachusetts USA) spectrometer. Specific surface areas measurements were performed on a Micromeritics ASAP 2010 instrument (ASAP 2010, Micromeritics, Shanghai (branch office), China) and analyzed by the BET method.

### 2.5. Adsorption and Degradation Experiments

The adsorption experiments of MB on as-fabricated samples were carried out by adding 0.01 g of samples into 50 mL dye aqueous solution (80 mg/L). The suspension was stirred in the dark for 4 h to ensure the dye and catalyst to reach adsorption-desorption equilibrium. The adsorption performance of sample was calculated according to Equation shown as following:

Adsorption capacity (mg/g) = (C_0_ − C_e_) × (V/m) (1)

where q_e_ is equilibrium adsorption capacity of samples, mg/g; C_0_ is initial dye concentration and C_e_ is the equilibrium adsorption concentration of dye concentration, mg/L; V is the volume of the solution, L; and, m is the mass of samples, g.

Adsorption = [(A_0_ − A_1_)/A_0_] × 100% (2)

where A_0_ is an initial absorbance of MB, A_1_ is an absorbance measured at a definite time.

The photocatalytic activity of the samples was evaluated by photo degradation of MB solution with a cooling-water-cycle system 0 A 300 W xenon lamp (FSX–300, NBeT Group Corp., Beijing, China) with a 420 nm cut off filter was used as a visible light source. At specific intervals of illumination, 2 mL of suspension was sampled and centrifuged to obtain clear solution. Decreases in the concentrations of MB solution were measured at 665 nm via a UV–vis spectroscopy (UV 2600, Shimadzu, Shanghai (branch office), China). The photocatalytic degradation was estimated from Equation (3) [30].

Photocatalytic degradation = [(A_2_ − A_t_)/A_2_] × 100% (3)

where A_2_ is an initial photocatalytic degradation of dye and A_t_ is an absorbance measured at a definite time.

### 2.6. Main Kinetics Models

Two main kinetics models were used to investigate the dye adsorption and photodegradation kinetics on the AKB, PBM, and BKBC. The expressions of models were presented, as following:

- Pseudo-first order model
  (4) $$\;\;\log\left(q_{e}-q_{t}\right)=\log\left(q_{e}\right)-\frac{\mathrm{KT}\cdot t}{2.303\;}\;$$
  q_e_ and q_t_ are, respectively, the amounts of MB (mg/g) adsorbed at equilibrium and at time t. K_1_ is the rate constant of the pseudo-first order (min^−1^).
- Pseudo-second order model
  (5) $$\;\frac{t}{q_{t}}=\frac{1}{\left(K_{2}q_{e}^{2}\right)}+\frac{1}{q_{e}}t\;\;$$
  q_e_ and q_t_ are, respectively, the amounts of MB (mg/g) adsorbed at equilibrium and at time t. K_2_ is the rate constant of the pseudo-second order (min^−1^).

### 2.7. Photodegradation Formula

(6) $$\ln(\frac{C_{0}^{\prime }}{C_{t}^{\prime }})=K_{\mathrm{app}}t$$

where $C_{0}^{\prime }$ is the initial photodegradation concentration of MB and $C_{t}^{\prime }$ is the photodegradation concentration of MB under light irradiation at the time, t.

In this study, all of the texts results were indicated as mean ± standard deviation. Moreover, every experimental treatment would obtain an average calculated from three replicates. The analysis of variance of data was adopted and it would be acceptable when p was less than 0.05 [31].

## 3. Results and Discussion 

### 3.1. Characterization of Activated Kelp Biochar Nanomaterials

**Figure 1:**
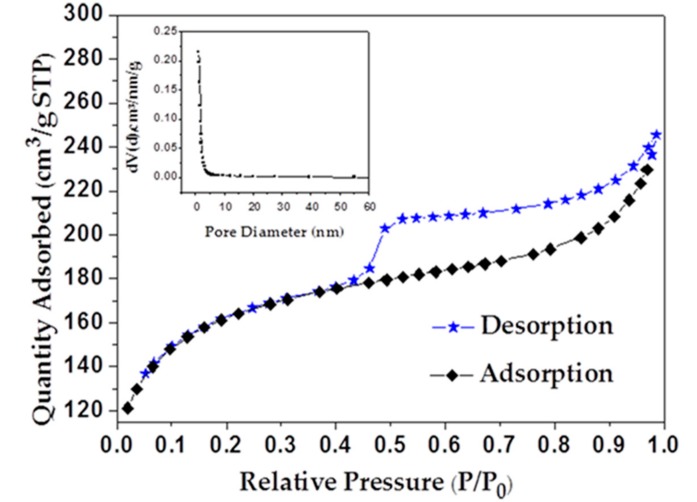
Curves of N_2_ sorption isotherms and pore size distribution (inserted) calculated from the density functional theory for activated kelp biochar (AKB). (* STP represents a temperature of 0 °C (273.15 K) and a pressure of 101.325 KPa (1 standard atmosphere, 760 mm Hg). P represents the vapor pressure on the meniscus with a temperature T and a radius of curvature r; P_0_ represents the saturated vapor pressure on a flat liquid of temperature T).

As shown in Figure 1, nitrogen adsorption isotherm and the pore size distribution (PSD) of AKB are exhibited that AKB is type IV isotherms with a hysteresis loop at P/P_0_ > 0.4. The capacity of nitrogen adsorption increases slowly as relative pressure less than 0.05, which suggests the presence of both micropores and mesopores. The analysis of specific surface areas and pore volumes from nitrogen adsorption isotherm data are calculated as 507.177 m^2^/g and 0.3797 cm^3^/g, respectively.

Figure 1 shows pore size distribution (PSDS) mainly in the range of 1.1–15 nm, which mostly consists of mesopores. This indicates that, in this experiment, as-prepared AKB has a wide range of specific surface areas. This high and uniform porosity may provide abundant adsorption sites and help highly enhance the photocatalytic activity of pollutant decomposition. 

Some of transition metal elements are detected by ICP analyses and listed in Table 1. Results of element analysis of AKB via ICP are shown that Ca (1.07 × 10^5^ mg/kg), Mg (1.49 × 10^4^ mg/kg), Al (6.15 × 10^4^ mg/kg), Sr (2.38 × 10^3^ mg/kg), and K (2.61 × 10^3^ mg/kg) are major cations in AKB. It has been found that the total contents of transition metal elements are more than 698.1 mg/kg, Fe_2_O_3_, MnO_2_, Mn_3_O_4,_ Mn_5_O_8_, or other metal oxide are likely to exist in AKB, which might be beneficial to the generation of photoinduced electron–hole pairs [32,33,34]. As illustrated in Table 2, some non-metallic elements of AKB are also detected and measured via an elemental analyzer, which indicates to consist of abundant carbon content in AKB.

**Figure 2:**
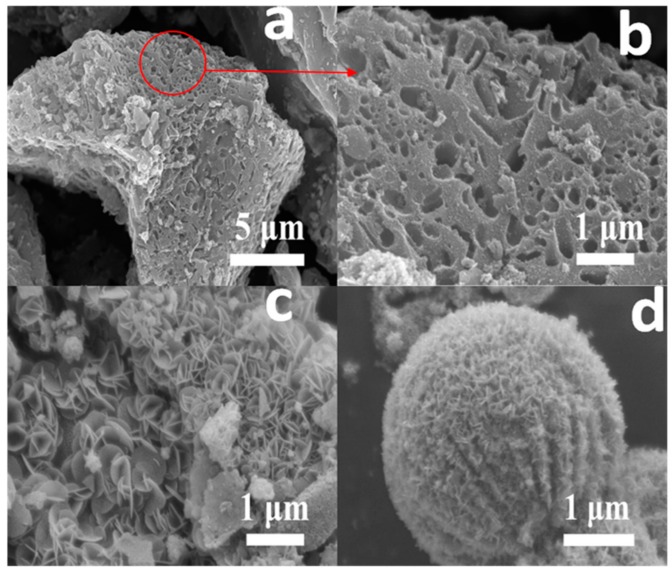
Scanning electron microscopy (SEM) images of AKB with a higher magnification (**a**,**b**); (**c**) Bi2MoO6/AKB nanocomposite (BKBC); and, (**d**) pure Bi2MoO6 (PBM).

The surface morphologies of AKB, PBM, and BKBC composite are investigated by SEM, as shown in Figure 2. AKB possesses abundant pore structures. A large number of pores with an average diameter of 200–300 nm are shown in Figure 2b. Meanwhile, BET data above show the main distribution of pores in the range of 1.1–15 nm for AKB. Figure 2c shows the SEM images of BKBC. It can be observed that a large quantity of Bi_2_MoO_6_ nanosheets grow on the surface of activated kelp carbon with well dispersity, which indicates that the successful formation of BKBC composite. As a comparison, pure Bi_2_MoO_6_ crystals synthesized without the presence of AKB appear to be microsphere, as shown in Figure 2d. Although some nano-flakes emerge on the surface of microspheres of Bi_2_MoO_6_, this aggregation form of Bi_2_MoO_6_ can only result in less efficiency of its photocatalysis.

**Figure 3:**
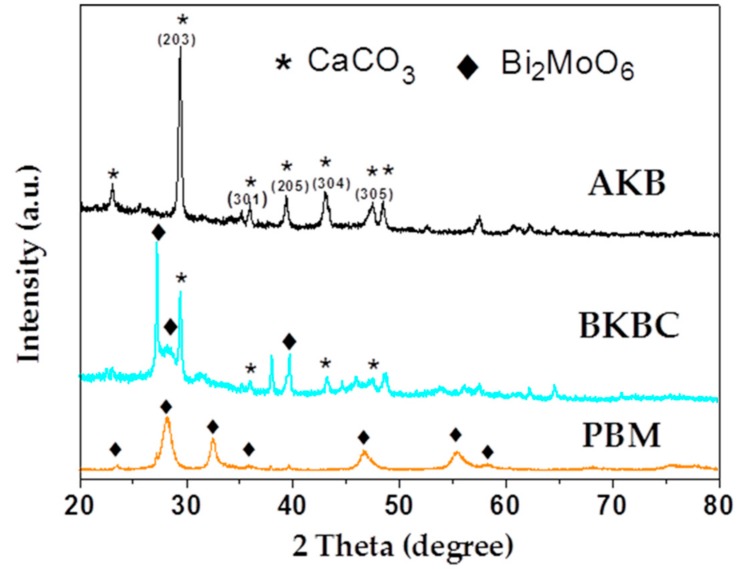
XRD patterns of AKB, BKBC, and PBM.

In Figure 3, the XRD pattern was employed to analyze the crystal structure and phase analysis of as-fabricated samples. As for the pattern of PBM, the observed diffraction peaks located at about 23.3°, 28.1°, 32.3°, 35.8°, 46.7°, 55.3°, and 58.2° could be perfectly indexed to the (111), (131), (200), (151), (062), (331), and (191) crystal planes of orthorhombic Bi_2_MoO_6_ (JCPDS76-2388), respectively. 

In the XRD pattern of AKB, the diffraction peaks were mainly concentrated between 20° and 60°, the peaks that were located at 29.5°, 35.2°, 39.5°, 43.0°, and 47.6° could be indexed to the (203), (301), (205), (304), and (305) planes of carbon black (JCPDS22-1069), indicating that the as-prepared kelp biochar nanomaterial might be an excellent electrical conductor with effective photocatalytic performance, which could promote the migration rate of photogenerated carriers and decelerate the electron-hole recombination [35,36]. Two main peaks that were located at 2θ = 23.0° and 29.34°, which could be indexed to calcite according to the JCPDS database No.81-2027. Therefore, the main components of AKB were proven to be CaCO_3_, and amorphous carbon, i.e., active carbon. In addition, it was surprising that the XRD diagram of AKB was in good agreement with our above assumptions. The obtained XRD pattern peaks located at 35.1°, 39.3° and 48.6° could derive from the hexagonal crystal structure of Fe_2_O_3_ (JCPDS40-1139) [37], and two evident diffraction peaks at 2θ = 35.3° and 47.6° assigned to (002) and (401) planes in the monoclinic phase of Mn_5_O_8_ (JCPDS39-1218) might suggest the presence of Mn_5_O_8_ components, and it could make a contribution to photocatalysis of AKB [38].

For BKBC composite, the primary diffraction peaks of Bi_2_MoO_6_ and CaCO_3_ could be clearly noticed, indicating that part of Bi_2_MoO_6_ was successfully combined with AKB. Moreover, the intensity of diffraction peaks of Bi_2_MoO_6_ exhibited a relative increase with the increase of Bi_2_MoO_6_ content.

**Figure 4:**
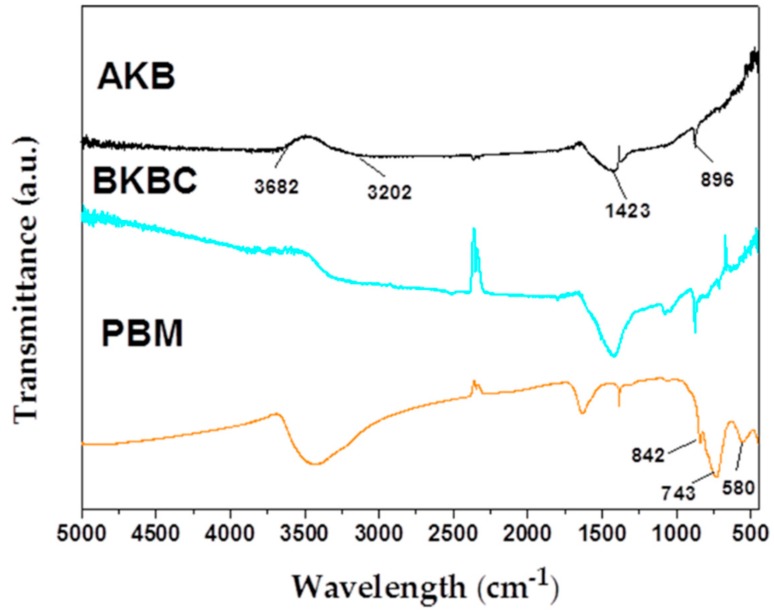
FT-IR spectra of AKB, BKBC, and PBM.

FT-IR analyses are carried out to investigate the chemical structures and components of as-prepared samples, and Figure 4 shows the FT-IR spectra of AKB, PBM, and BKBC. The FT-IR spectrum of AKB exhibits a broad absorption around 3200–3600 cm^−1^, due to the stretching vibration of –OH groups. The typical absorption behavior of a wide band appearing at 1400–1600 cm^−1^ is mainly attributed to π = π stretching vibration of benzene ring, indicating that the reduction of non-polar aliphatic functional groups in biochar and the increase of aromatization degree. A weak band appearing at 896 cm^−1^ may be ascribed to the =C–H stretching vibration. Therefore, there are an abundant of various functional groups, such as –OH and =C–H, presented on the surface of AKB, which may contribute to the high adsorption capacity of AKB. The spectrum of PBM contains some strong characteristic bands, such as Mo = O (~842 cm^−1^) stretching vibration and the absorption at 743 cm^−1^ is attributed to the tetrahedral stretching vibration of Mo(VI)–O groups. However, a band at 580 cm^−1^ may be due to the bending mode of MoO_6_. For the as-prepared BKBC composite, their characteristic absorption bands are all observed from the spectrum of BKBC, indicating the presence of Bi_2_MoO_6_ nanosheets grown on AKB nanomaterials. This observation is consistent with the analysis results of SEM results (Figure 2) and XRD patterns (Figure 3).

**Figure 5:**
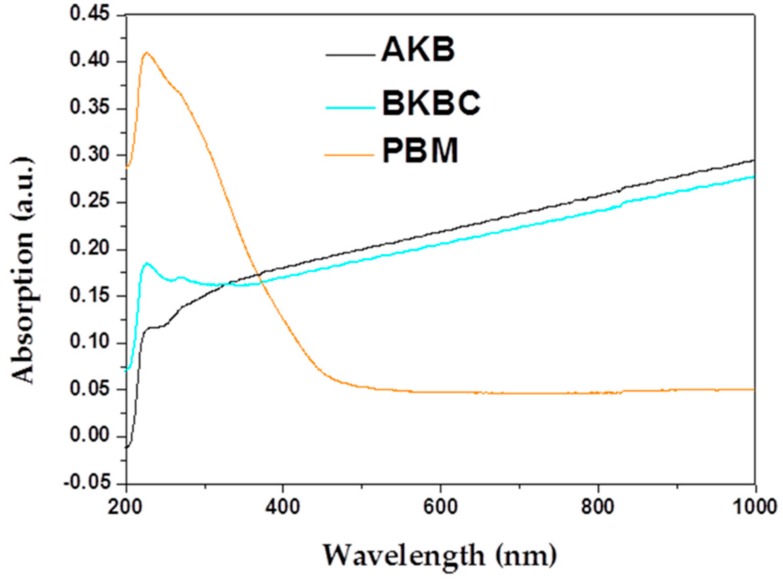
UV-Vis diffuse reflectance spectra of AKB, BKBC, and PBM.

The difference in optical absorption could highly affect photocatalytic performance of samples. As illustrated in Figure 5, the UV–vis diffuse reflectance spectra of AKB, PBM, and BKBC are various with each other attributed to their different photoabsorption properties from UV light region to visible light region. The absorption edges of PBM are located at 478 nm exhibiting a weak absorption in the visible light range, and its band gaps (E_g_) are calculated to be 2.59 eV. When compared with PBM, the BKBC composite shows significantly enhanced absorbance in the visible-light region (λ > 400 nm). As shown in Figure 5, AKB exhibits an intense absorption in the visible light range, indicating that AKB itself may have an effective photocatalytic activity that is attributed to its internally contained transition metal oxide, as well as its high active carbon content.

### 3.2. MB Sorption and Degradation

#### 3.2.1. Adsorption Kinetics

**Figure 6:**
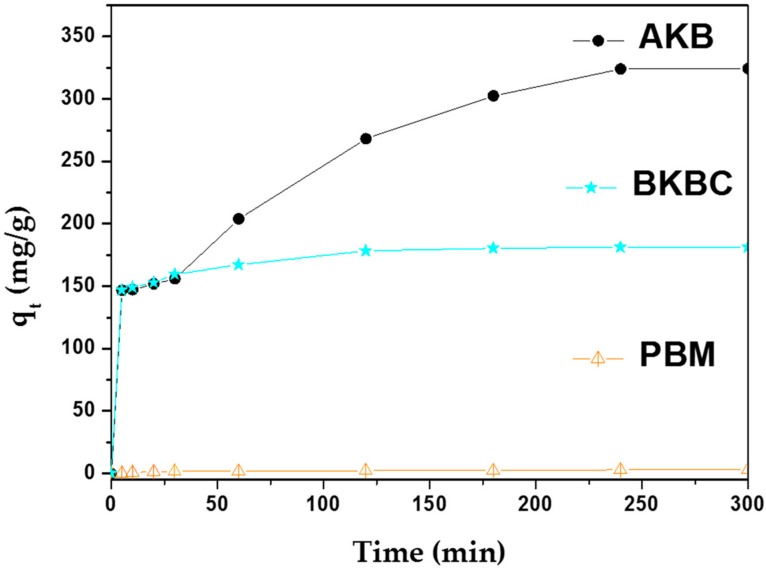
Effect of contact time on methylene blue (MB) adsorption by AKB, BKBC, and PBM.

In order to compare the adsorption capacity of MB on different samples at the following conditions: 50 mL of dye aqueous solution at a relatively high concentration of 80 mg/L, 0.01 g of adsorbent, and an adsorption contact time of 4 h (as shown in Figure 6).

Adsorption experiments are also implemented to reveal the mechanism of MB adsorption on AKB, PBM, and BKBC composite after reached equilibrium for 4 h without exposure to visible light illumination. The maximum adsorption capacity of different samples to MB is illustrated in Table 3. As shown in Table 3, an adsorption capacity of 324.1 mg/g can be obtained by AKB, which is higher to the all other adsorbents [39].

**Figure 7:**
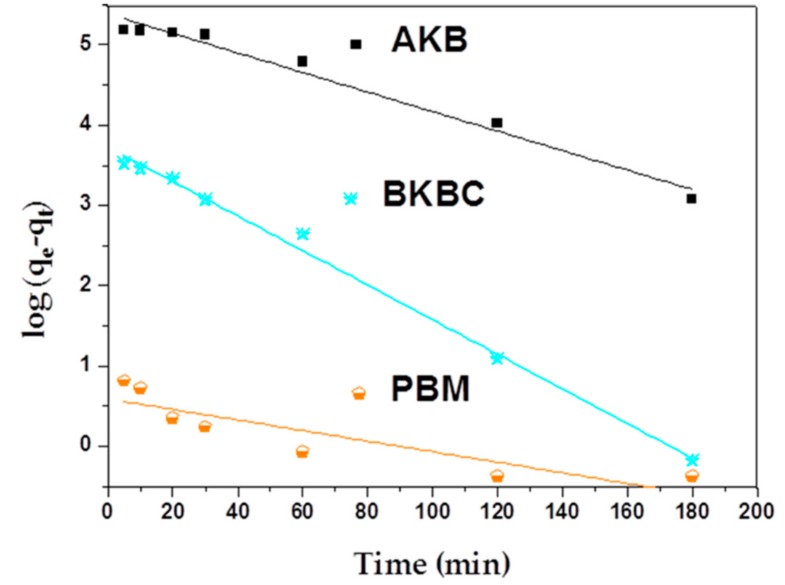
Pseudo-first order kinetics plots for adsorption of AKB, BKBC, and PBM.

**Figure 8:**
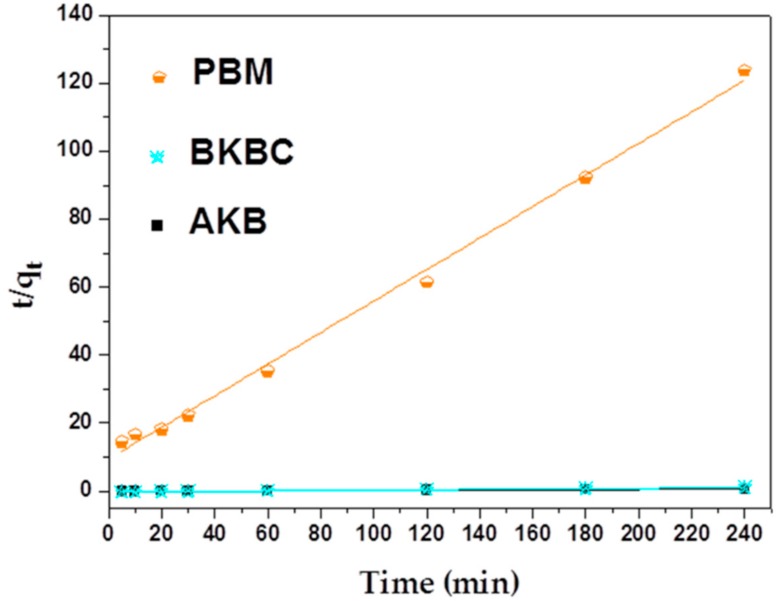
Pseudo-second order kinetics plots for adsorption of AKB, BKBC, and PBM.

As shown in Figure 7 and Figure 8, a pseudo-second order model complies with a real adsorption process well. The maximum adsorption capacity of MB calculated by the reference of pseudo-second equations is approximately equal to the equilibrium adsorption capacity (as shown in Table 3). Moreover, the adsorption kinetics results are presented in Table 4. It can be seen that the calculated values of regression coefficients of pseudo-second order model are much higher than that of pseudo-first order model, which is very close to 1. Therefore, the sorption process involves chemical reactions while in view of the assumption of pseudo-first-order model [40], and chemical interactions between adsorbent and adsorbate include the sharing and the transfer of electron pairs.

#### 3.2.2. Photocatalytic Activity

**Figure 9:**
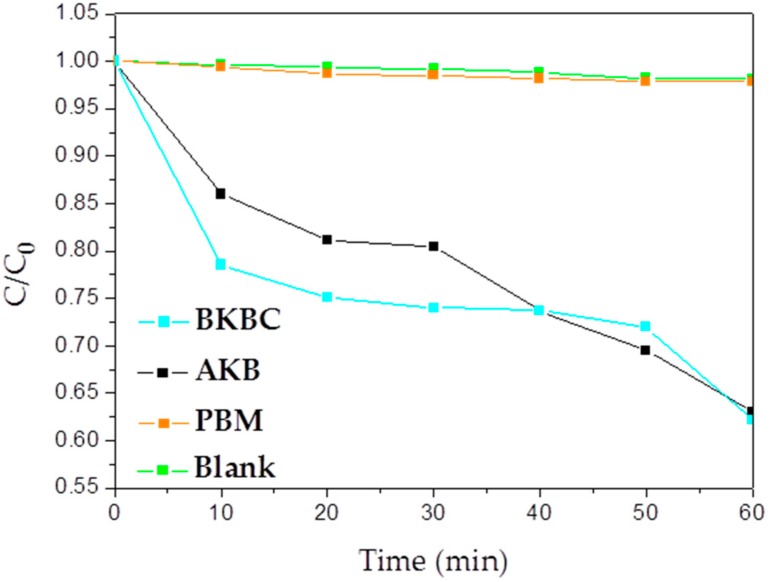
Degradation efficiency of MB for AKB, BKBC, blank, and PBM.

After the adsorption equilibrium of MB on as-prepared samples, the photodegradation studies are implemented to evaluate the photocatalytic efficiency of as-prepared samples under visible light irradiation. As seen in Figure 9, the degradation of MB is extremely slow with PBM, which is even not much difference from blank control samples. The low degradation activity of PBM may be due to the excessive high concentration of dye or insufficient reaction time. Its limited contact with dye is because of the less available active sites and the intermolecular agglomeration of pure Bi_2_MoO_6_. The photocatalytic activity of BKBC is better than PBM and AKB, which may be attributed to the addition portion of Bi_2_MoO_6_ component as its large quantity of nanosheets uniformly growing on the surface of AKB and also as it is one of the excellent photocatalysts for the degradation of organic compounds under visible light irradiation. BKBC exhibits an enhanced photocatalytic activity that is much higher than PBM due to the synergetic effects of effective absorption as well as heterojunctions that are constructed between Bi_2_MoO_6_ nanosheets and AKB, which is attributed to the existence of abundant active carbon and various transition metals oxides in kelp biomass and also can further prevent the recombination between photoelectrons and holes. For comparison, blank tests are carried out at the same identical conditions, except the absence of either irradiation or photocatalyst.

**Figure 10:**
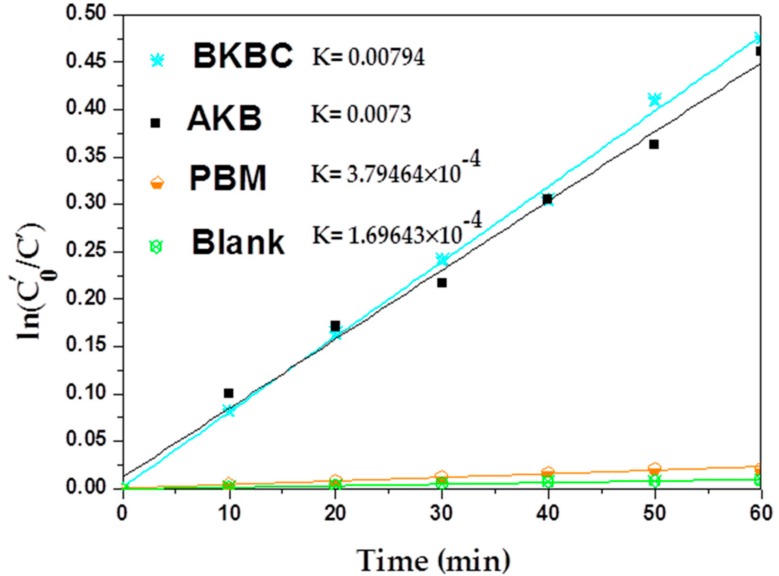
Kinetic process of MB degradation for AKB, BKBC, PBM, and blank.

As shown in Figure 10, the experiment data of photocatalytic degradation fit well with a first-order model. For all samples, their apparent rate constant values for the photocatalytic degradation of MB are represented in Figure 10. When compared with PBM, the degradation rate of BKBC has been a dramatic change increasing from 0.00038 min^−1^ to 0.0079 min^−1^, indicating that AKB as supporter could promote the progress of photocatalytic reactions. 

In Figure 10, BKBC exhibits the best photocatalytic performances among them, while the percent of MB degraded (out of total MB) is 16.11%. The enhanced degradation efficiency may be attributed to the synergetic effects of effective absorption as well as heterojunctions constructed between Bi_2_MoO_6_ nanosheets and AKB. It is speculated that the Bi_2_MoO_6_ can be excited to produce electrons-holes that are attributed to the utilization of solar energy. However, the Kapp value of BKBC (as shown in Figure 10) is somehow less than other semiconductor composites such as Bi_2_MoO_6_/graphene with K = 0.0136 min^−1^ [41] and Bi_2_MoO_6_/diatomite, 0.05892 min^−1^ [42], Bi_2_MoO_6_/Fe_2_O_3,_ 0.08586 min^−1^ [43], Bi_2_MoO_6_/C_3_N_4_, 0.0792 min^−1^ [44], and Bi_2_MoO_6_/CNTs/g-C_3_N_4_, 0.0078 min^−1^ [45], as listed in Table 5. This phenomenon may be caused by the excessive high concentration (80 mg/L) of MB. Because activated kelp biochar has a very strong capacity of adsorption towards MB. There will be no MB available for degradation of BKBC as a low concentration of MB used in the system. In general, when the pollutant concentration exceeded to certain value, the catalytic activity was reduced to some extent [36]. When more dye molecules were adsorbed by BKBC, less active groups on the surface of catalyst would be available. There will be a strong competition among the reaction sites, leading to a decrease of the photocatalytic efficiency of BKBC. Moreover, the content of Bi_2_MoO_6_ added in BKBC is only a small amount when compared with others in literatures, as shown in Table 5, with the mass ratio of Bi_2_MoO_6_ to activated kelp biochar of 1:4. 

**Figure 11:**
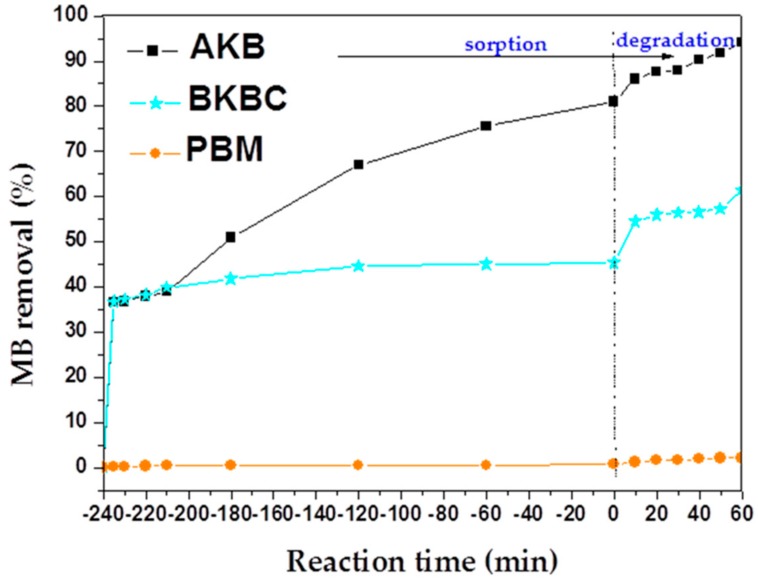
Adsorption and photodegradation progress of MB (80 mg/L) by AKB, BKBC, and PBM as a function of time. The photocatalytic degradation was initiated after sorption for 4 h.

As exhibited in Figure 11, AKB also presents an excellent sorption capacity and a high catalytic activity, while the percent of MB degraded (out of total MB) is 13.085% after visible light illumination for 1 h, indicating that AKB may have an excellent photodegradation activity due to the existence of various transition metals oxides and abundant active carbon in AKB biomass, which is suggested by the results of elemental analysis and XRD patterns. The values of MB removal for as-prepared samples are presented in Figure 11, the highest removal efficiency approximately to 94.11% is obtained by AKB due to its both excellent sorption and degradation properties for organic pollutants. As illustrated above, in Figure 5, AKB exhibits an intense absorption in the visible light range, which indicates that AKB itself may have an effective photocatalytic activity that is attributed to its internally contained transition metal oxide as well as its high active carbon content, the abundant active points resulting from high specific surface areas. Therefore an excellent adsorption and photodegradation performance of AKB has been verified and demonstrated in this work.

## 4. Conclusions

Novel mesoporous nanomaterials AKB and BKBC with excellent adsorption and photodegradation ability are successfully prepared and synthesized. In this work, BKBC exhibits an enhanced photocatalytic activity due to the synergetic effects of effective absorption as well as heterojunctions that are constructed between Bi_2_MoO_6_ nanosheets and AKB. In addition, AKB presented the higher efficiency of MB removal, even up to 94%, which is attributed to abundant active sites and high specific surface areas, and the transition metal oxides contained could also promote to the evident enhancement of degradation efficiency. Consequently, the inexpensive AKB may have its great potential for the application of environmental remediation.

## Figures and Tables

**Table 1:** Main metal elements of AKB obtained by inductively coupled plasma (ICP), mg/kg.

Metal Elements	Cr	Er	Fe	Mn	Ni	Ti	Y
AKB	37.9	17.9	510	72.1	3.4	8.8	15.3
Metal elements	**Sr**	**Al**	**Ca**	**K**	**Mg**	**Zn**	**Cu**
AKB	2.38 × 10^3^	6.15 × 10^4^	1.07 × 10^5^	2.61 × 10^3^	1.49 × 10^4^	37.8	11.1

**Table 2:** Some non-metallic elements of AKB obtained by elemental analyzer (mass, %).

Non-Metallic Elements	C	H	N
AKB	59.81	2.30	2.41

**Table 3:** Maximal adsorption capacity of MB on as-prepared samples.

Adsorbents	q_e_ (mg/g)
PBM	2.65
BKBC	181.1
AKB	324.1

**Table 4:** Adsorption kinetics parameters of MB on samples.

Adsorbents	Pseudo-First Model			Pseudo-Second Model		
	q_e_.Cal (mg/g)	k_1_ (min^−1^ )	R^1^	q_e_.Cal (mg/g)	k_2_ (g mg^−1^ min^−1^)	R^2^
AKB	217.58	0.01214	0.97497	343.64	1.18603 × 10^−4^	0.97796
BKBC	42.11	0.02153	0.99412	183.49	1.57566 × 10^−3^	0.9997
PBM	1.83	0.00657	0.75445	2.15	0.02290	0.99582

**Table 5:** Kapp values of different photocatalyst composites in literatures and this work.

Catalyst	Dye	Dye (mL)	Dye Conc. (mg/L)	Catalyst Content (g)	Reaction Time (min)	K_0_ * (min^−1^)	K * (min^−1^)	Ref.
**Bi_2_MoO_6_/graphene**	MB	100	0.01	0.05	120	0.0037	0.014	[41]
**Bi_2_MoO_6_/diatomite**	RhB	40	4	0.015	60	0.019	0.059	[42]
**Bi_2_MoO_6_/Fe_2_O_3_**	RhB	50	5	0.03	60	0.015	0.086	[43]
**Bi_2_MoO_6_/C_3_N_4_**	RhB	50	10	0.02	50	0.0027	0.079	[44]
**Bi_2_MoO_6_/CNTs/*g*-C_3_N_4_**	2,4–DBP	250	20	0.25	120	0.0013	0.0078	[45]
**AKB**	MB	50	80	0.01	60	0.00038	0.0073	This study
**BKBC**	MB	50	80	0.01	60	0.00038	0.0079	This study

* K_0_ represents the Kapp value of pure Bi_2_MoO_6_, K represents the Kapp value of different photocatalyst composites.
